# Assessing the feasibility of a co-produced peer-group intervention for supporting wellbeing during the transition to adulthood among autistic 16–25-year-olds (ATAG): a randomised controlled feasibility trial

**DOI:** 10.1016/j.eclinm.2026.103859

**Published:** 2026-04-07

**Authors:** Kate Cooper, Rachel A. VanDaalen, Annabel Burnley, Leon Allain, Bryony Beresford, Laura Crane, Labeebah Islaam, Maximiliano Vazquez Morales, Lucy Portway, Benjamin Redmayne, Ailsa Russell, William Mandy

**Affiliations:** a Research Department of Clinical, Educational, & Health Psychology, University College London, London, United Kingdom; b Department of Psychology, Centre for Applied Autism Research, University of Bath, Bath, United Kingdom; c Department of Psychiatry, University of Oxford, Oxford, United Kingdom; d Oxford Institute of Clinical Psychology Training and Research, University of Oxford, Oxford, United Kingdom; e Ambitious about Autism, London, United Kingdom; f School for Business and Society, University of York, York, United Kingdom; g Social Policy Research Unit, University of York, York, United Kingdom; h Centre for Research in Autism and Education (CRAE), IOE, UCL's Faculty of Education and Society, University College London, London, United Kingdom; i Department of Disability, Autism Centre for Education and Research (ACER), Inclusion and Special Needs, School of Education, College of Social Sciences, University of Birmingham, United Kingdom; j Population Health Sciences, University of Bristol, Bristol, United Kingdom; k Bristol Trials Centre, University of Bristol, Bristol, United Kingdom

**Keywords:** Feasibility study, Randomised controlled trial, Autism, Wellbeing, Transition to adulthood

## Abstract

**Background:**

Autistic people, including those without intellectual disability, face challenges during the transition to adulthood, and experience poor adult outcomes. We conducted a feasibility trial of a novel co-produced peer group intervention to support the transition to adulthood in autistic people.

**Methods:**

Autistic 16- to 25-year-olds were recruited to this feasibility randomised controlled trial via autism charities and research databases in England and Wales. Participants needed sufficient literacy abilities to be able to access the intervention materials. Participants were randomly assigned (1:1), via an online randomisation service, stratified by age (16–17 and 18–25 years) to the peer group intervention or care as usual (CAU). Participants in both arms could access CAU, which was the routine care offered to autistic 16–25 year-olds in their region, and so varied by participant. The intervention comprised six hour-long online peer group psychoeducation sessions to improve autism knowledge, autism social identity, and self-advocacy skills. The main outcomes of this feasibility trial were as follows: recruitment and retention rates; the acceptability of randomisation and outcome measurement procedures; CAU accessed by participants; acceptability of the interventions; and clinical outcome measure variances. Clinical outcome measures were collected at baseline, and 8-, 16-, and 24-weeks after randomisation. The primary clinical outcome measure was wellbeing at 16-weeks, using the Warwick Edinburgh Wellbeing Scale [WEMWBS], and all randomised participants were included in the analyses. A co-produced qualitative study investigated trial experiences. The trial is registered with the International Standard Randomised Controlled Trial Number [ISRCTN] registry, ISRCTN10513626.

**Findings:**

Seventy participants were recruited between 1st November 2023 and 10th June 2024 (*n* = 35 per arm). At 16-weeks, the primary clinical outcome measure (wellbeing score) was completed by 74% (*n* = 26) of the intervention group and 91% (*n* = 32) of the CAU group. In terms of CAU, across both arms, participants were most likely to have used medications and primary and community care. Adverse events were comparable between arms (intervention group: 6, CAU group: 7). Qualitative feedback indicated that both study procedures and the intervention itself were acceptable.

**Interpretation:**

A full trial appears to be feasible. Limitations including retention in the intervention arm and limited sample representativeness would need to be addressed for a full trial.

**Funding:**

National Institute of Health and Care Research (NIHR) Research for Social Care and Autistica.

**Research in context**

**Evidence before this study**

We searched PsycInfo on 9th May 2025 using the search terms (Autism) AND (Post-Diagnostic). This resulted in 42 articles, of which 14 were relevant to this project. From the citations in these papers, we identified a further 23 articles of relevance. Alongside this, we consulted a recent systematic review of post-diagnostic support for autistic adults. There are no existing feasibility randomised controlled trials (RCT) in this area, but there have been feasibility and pilot studies (without randomisation) of psychoeducation and group interventions for autistic adults and young adults. These studies indicate the promise of such interventions, but there are no published full trials of such interventions.

**Added value of this study**

To our knowledge, our study is the first co-produced feasibility RCT of a peer group intervention for autistic people during the transition to adulthood. We also conducted a co-produced qualitative study that aimed to understand experiences of trial participation, including experiences of randomisation, to support the design of future trials in this area.

**Implications of all the available evidence**

Our findings suggest that autistic young people aged 16–25 years are willing to take part in RCTs and found trial procedures, including randomisation and outcome measurement procedures, broadly acceptable. The group intervention was also reported as being acceptable. We provide suggestions for designing trials with autistic young people based on feedback on our co-produced methods in this feasibility RCT.

## Introduction

Autism is a neurodevelopmental condition characterised by differences in social communication and interaction, alongside restricted and repetitive differences in behaviours, interests and activities. Autistic people, including those without intellectual disability (ID), have poor outcomes compared to non-autistic people. These poor outcomes include mental and physical health difficulties, low quality of life, social isolation, unemployment/underemployment, homelessness, and premature mortality.^1^, ^2^, ^3^, ^4^ Autistic people without ID are a high-risk group for poor adult outcomes since their needs are deemed not high need enough for autism-specific services, yet too complex for standard mental healthcare services.^5^

In this paper we focus on the needs of autistic individuals without ID during the transition to adulthood (16–25 years). This is a challenging time for all young people, and especially autistic individuals, due to changing demands in terms of independence, alongside a reduction in support from family and education, health and care services.^6^, ^7^, ^8^ Parents/carers also experience significant personal challenges when their autistic children struggle in emerging adulthood.^9^^,^^10^

There is no direct evidence on the effectiveness of interventions supporting autistic people during the transition to adulthood.^11^ This stark lack of evidence-based support has a substantial negative effect on their wellbeing, functioning and life chances, and on their parents’ wellbeing.^9^^,^^11^ Further, the lack of completed clinical effectiveness trials in this field means that there is limited information to draw on to design and conduct trials that are acceptable to the autistic community, and that are feasible.

Self-management, service engagement and positive autism identity can contribute to better outcomes for autistic adults according to observational studies and a systematic review of university transitions.^12^, ^13^, ^14^ A co-production partnership with our team and autistic young people, led by our partner charity Ambitious about Autism, resulted in a co-produced group intervention to support autistic people without ID during the transition to adulthood. The co-production process has been reported in a separate paper.^15^ Co-production is critical in this research field where autistic people have routinely been dehumanised, and their lived experience has been overlooked.^16^

As a step towards understanding whether such interventions are effective in supporting autistic young people in the transition to adulthood, we conducted a feasibility randomised controlled trial allocating participants at a 1:1 ratio to either the new group intervention or care as usual. We also recruited carers to a carer impact study and we conducted a co-produced qualitative study.

Our objectives were as follows:

1. Assess the rates of recruitment and retention to inform the design of a full-scale randomised controlled trial (RCT).
2. Assess the acceptability of trial procedures to participants.
3. Characterise care as usual (CAU).
4. Assess the acceptability of the group intervention and CAU.
5. Calculate outcome measure variances for use in a power calculation for a full trial.

## Methods

### Study design

We conducted a two-arm parallel feasibility RCT and report our findings in adherence with the Consolidated Standards of Reporting Trials (CONSORT) reporting guidelines. The trial protocol was pre-registered (ISRCTN10513626) and published.^17^ Following consultation with our Patient and Public Involvement (PPI) groups and the trial steering group, the protocol was modified (please see Supplementary Table S1) through two ethics amendments, changing: participant payments, questionnaire wording, treatment dose (which was changed from four to three sessions on advice from the Trial Steering Committee), and clarifying risk protocols. We also recruited carers (when nominated by consented participants) to a carer-impact study. Finally, we conducted a co-produced qualitative study to understand participant, carer, and facilitator views on trial participation and the interventions.

Participant recruitment was via United Kingdom (UK) charities, e.g. Ambitious about Autism, National Autistic Society and Autistica, and lists of potential research participants, e.g. at the Centre for Applied Autism Research at the University of Bath. We also recruited from one pilot National Health Service (NHS) autism service as a participant identification centre (PIC) to understand the feasibility of recruitment in NHS settings.

The study received ethical approval from the Health Research Authority and Wales Research Ethics Committee (REC) 5 (Reference: 23/WA/0113).

We are a neurodiverse research team that includes four public members; three autistic young people and one Programme Manager at our partner charity, Ambitious about Autism. Ten autistic young people, together with Ambitious about Autism and academic partners, co-designed the intervention^15^ and trial design, including outcome measures. Two autistic young people were included in the study leadership team. Five autistic young people were trained as facilitators to co-deliver the intervention, and two acted as co-researchers on the qualitative study. All autistic young people were paid for their time and reimbursed for expenses. See Supplementary Table S2 for the Guidance for Reporting Involvement of Patients and the Public (GRIPP2) statement.

### Participants

We recruited autistic 16- to 25-year-olds. This age range was selected to represent the transition to adulthood. This age range aligns with service provision for young adults in the UK.

#### Inclusion criteria

• Aged 16–25 years at the point of randomisation.
• A diagnosis of autism from a qualified professional who could plausibly have been trained in conducting autism diagnostic assessments (e.g. educational psychologist; paediatrician). Participants had to provide evidence (i.e. a clinic letter) during the eligibility assessment.

#### Exclusion criteria

• Participants who reported that they had received any autism post-diagnostic psychoeducational support from a professional over the past 12 months. This was defined as any support from a professional outside of the diagnostic assessment appointments about what an autism diagnosis means, how autism may affect the individual, and how to find support as an autistic adult.
• Risk of harm to self that would mean that a group intervention was not clinically appropriate, assessed using item 9 of the Patient Health Questionnare-9 (PHQ-9; which addresses thoughts of suicide and/or self-harm) and followed up by a clinician.
• Risk of harm to others such that group participation was not appropriate. This was assessed through a series of questions about the individual's school exclusion and forensic history and was followed up by a clinician when necessary.
• English, non-English and Welsh literacy levels such that the intervention materials were inaccessible without reasonable adjustments.

All participants were invited to nominate a carer, who was invited to enrol as a participant in our carer impact study. Facilitators of the group intervention were also recruited to participate in the qualitative study.

Individuals who were interested in participating were directed to an online expression of interest form. Potentially eligible individuals were invited to a baseline assessment and follow-up questions were used to establish eligibility as required. If they met eligibility criteria and provided written consent, they were enrolled. Carers and facilitators were also asked to provide fully informed consent. Prior to randomisation, the primary outcome measure (wellbeing) was administered a second time to those whose baseline assessment was more than two weeks before the start of the next groups. Randomisation was conducted within two weeks of the next group start date and communicated with the participant by email.

### Randomisation and masking

The randomisation sequence was generated by the secure online randomisation service Sealed Envelope™. Randomisation was stratified by age (16–17 or 18–25) and conducted in random permuted blocks of four. Participants were randomised on a 1:1 ratio to the intervention arm or CAU (control arm).

The Chief Investigator (or authorised delegate) signed into the secure online randomisation system, entered the individual's unique study identification number and age. They then received from Sealed Envelope the code that allocated the participant to either UYDY or CAU. The research team informed the individual of their allocation and securely shared the contact details of those allocated to UYDY to the team facilitating the groups. The unblinded randomisation code was held by selected members of the Trial Management Group (TMG).

Participants were aware of their group allocation. The TMG was masked as to the allocation of treatment group, except for members of staff involved in data management, trial facilitators and supervisors of trial facilitators. The success of this masking was not assessed.

### Procedures

The co-designed intervention was named ‘Understanding You, Discovering You' (UYDY), but following post-feasibility study co-design work with autistic young people, the intervention will be renamed ‘Navigate’. The original co-design process produced six sessions with facilitator guides, slides, and training materials.^15^

UYDY is a six-session, weekly, hour-long online peer-group psychoeducation program for autistic young people, which aims to increase autism knowledge, positive social identity, and self-advocacy for transition to adulthood. Groups of 7–10 young people were facilitated by an autistic person and two social care professionals (e.g. education staff, occupational therapists, support workers). Sessions combined didactic and experiential learning on: understanding the autism label; autistic strengths; problem-solving and goal-setting; disclosing diagnosis and rights; identifying needs and accessing services; and meeting social needs (see Supplementary Table S3).

Facilitators received 7 h of training on autistic young people's needs, service provision, communication adaptations, and online group delivery, plus monthly supervision. After each session they completed a Facilitator Record Form (FRF) documenting delivery and issues, including participant engagement.

Educational resources were also offered to participants’ nominated supporters (e.g. carer, partner, family member). This included written educational materials about the group session topics.

Young people in both arms of the trial could continue accessing their usual health and social care provision. CAU was measured via health and care service resource use questionnaires at baseline, 8-, 16-, and 24-weeks. Participants allocated to CAU could access the UYDY groups upon completion of their final follow-up.

All participants were asked to complete follow-up questionnaires at 8-, 16-, and 24- weeks after randomisation, with personalised reminders based on participants’ communication preferences.

### Outcomes

The main outcomes of this feasibility trial were as follows: recruitment and retention rates; the acceptability of randomisation and outcome measurement procedures; CAU accessed by participants; acceptability of the interventions; and clinical outcome measure variances.

Outcome measures were collected at baseline, 8-, 16- and 24- weeks post-randomisation. These are the anticipated outcome measures for a putative full trial, but since this is a feasibility study, scores on these measures are not the outcomes of the current study.

The primary clinical outcome measure (i.e. the anticipated primary outcome in a full trial) was the Warwick Edinburgh Wellbeing Scale (WEMWBS)^18^ at 16-weeks post-randomisation. This measure is validated for use in this age group, and with good internal consistency in autistic adults^19^ and good internal reliability in a sample of autistic young people aged 15–22 years.^20^ Items include “I've been feeling confident”, scored on a Likert scale from 1 “none of the time” to 5 “all the time”, and the total score is a sum of all 14 items, and the range is 14–70, with higher scores indicating better wellbeing. A change in three or more points is considered significant.

The secondary clinical outcome measures were the:

• Autism social identification measure,^13^^,^^21^ a measure of affiliation with the autistic community and with autistic identity, including items such as “I am glad to be autistic”. This 14-item scale has good reliability and construct validity in autistic adults^22^ and good reliability in autistic young people aged 15–22 years.^20^
• The 5-level EQ-5D (EQ-5D-5L) measure of quality of life^23^ for potential use in a future economic evaluation of the intervention versus CAU.
• Interpersonal Support Evaluation List^24^–short measured perceived social support.
• Short four-item University of California, Los Angeles (UCLA) loneliness scale (ULS-4)^25^ which measures experiences of distress due to a discrepancy between actual and desired social connection.
• A use of services questionnaire based on the Client Service Receipt Inventory (CSRI)^26^ to characterise CAU by thorough measurement of the additional health and social care services accessed by all participants during the study.

Carer impact was measured using the Adult Social Care Outcomes Toolkit-Carer (ASCOT-carer),^27^ a validated measure of social-care related quality of life for carers, and the WEMWBS wellbeing scale.^18^

A risk management standardised operating procedure was developed for use throughout the trial. This was implemented to ensure the safety of all participants who expressed significant distress during the research process or a desire or intention to harm themselves or others.

Adverse events (including serious adverse events) were recorded by the research team. These were identified when participants disclosed distress or difficulties in research appointments, in intervention sessions, or in their questionnaires, particularly the CSRI.

### Statistical analysis

The recruitment target was 70 participants in England and Wales. A sample size of 70, with 80% (*n* = 56) follow-up rates, was deemed sufficient to inform a sample size calculation for a full RCT and to evaluate the rates of recruitment and retention.^28^

The statistical analysis plan was prespecified, based on the trial protocol.^17^ The statistical analysis was conducted in Statistical Package for the Social Sciences (SPSS) Version 29 and a review of the analysis was conducted in Stata v18.0 We did not plan to assess potential effectiveness in this study since (a) we did not power the study for such analyses, and (b) we needed to answer preliminary questions about the feasibility of conducting a larger trial before doing so.

The data from all individuals who consented for each arm (n = 35 each) were included in the analyses.

To meet objective 1, assessing recruitment and retention, we calculated the percentage of those who were eligible and consented to be randomised. We calculated retention rates as percentages of the number of participants in each arm who completed follow-up measures at each time point.

For objective 2, about acceptability of trial procedures, we conducted reflexive thematic analysis of one-to-one interviews with young people, carers and facilitators.

For objective 3, we characterised CAU by calculating the proportion of participants in CAU who received each type of support.

For objective 4, we assessed intervention acceptability by calculating the percentage of those who completed the intervention, mean number of sessions completed and standard deviation, and by calculating the number of adverse events in each arm.

For objective 5, we assessed outcome measure variances with descriptive statistics, including means and standard deviations, calculated for each outcome measure by intervention group and timepoint. We also conducted reflexive thematic analysis.

The carer data were also analysed as described in objectives 1 and 5 above.

The study had a combined Trial Steering and Data Monitoring and Ethics Committee which included an independent statistician.

### Qualitative study

A co-produced qualitative study aimed to understand participant experiences of research participation, CAU, and UYDY for those who accessed this. All participants were asked if they consented to be interviewed at baseline and verbal consent was recorded during interviews.

Interviews were conducted with 12 (34%) autistic young people allocated to UYDY and 10 (29%) to CAU, seven (23%) parents/carers and eight (100%) UYDY facilitators, of whom five (63%) were autistic. Overall, we interviewed 37 participants, 74% of the qualitative study target sample size. We interviewed everyone who responded to interview invites: financial incentives were not offered for these interviews which may have contributed to the lower than anticipated take-up rate.

Autistic and non-autistic team members conducted interviews by video or audio-only calls. The co-produced topic guide covered: recruitment experiences; preference for intervention allocation; experience of CAU and UYDY; experience of follow-up assessments; improvements to UYDY; and increasing access and inclusion for under-represented groups. Interviews were audio recorded via Zoom.

Adaptations to interviews were made to communication method (e.g. using the chat function), environment (e.g. ensuring the individual had access to fidget toys where needed), and structure (e.g. having regular breaks where needed).

### Qualitative analysis

Interview recordings were transcribed, anonymised, checked for accuracy and imported into NVivo qualitative data analysis software. A team Reflexive Thematic Analysis was conducted from a critical realist standpoint, as defined by Braun and Clarke. Line-by-line coding was undertaken: first, all team members coded one transcript and discussed their approach to ensure consistency. Then transcripts were divided between team members, with regular meetings to consolidate similar codes and ensure consistency, with senior team members checking coding. Team theme development ensured that a diversity of viewpoints contributed to the final analysis. Two senior team members wrote the analysis and all team members suggested amendments. The team analysis was presented to the other co-authors and oversight groups for comment. Here, we present only the qualitative findings linked to research procedures and outcome measurements, and a separate paper focuses on experiences of UYDY and CAU. The qualitative findings linked to research procedures and outcomes (presented here) tended to follow mostly semantic, rather than latent, codes that were driven by participant responses. As participants' statements about research procedures were rather descriptive and less emotive and in-depth compared to statements about intervention experiences, the themes presented here are less interpretative in nature.

### Role of the funding source

The funder of the study had no role in study design, data collection, data analysis, data interpretation, or writing of the report. Authors had full access to the data in the study and KC and WM had final responsibility for the decision to submit for publication.

## Results

We recruited participants between 1st November 2023 and 10th June 2024, with the first participant consented on 5th December 2023 and the final participant consented on 10th June 2024.

**Fig. 1:**
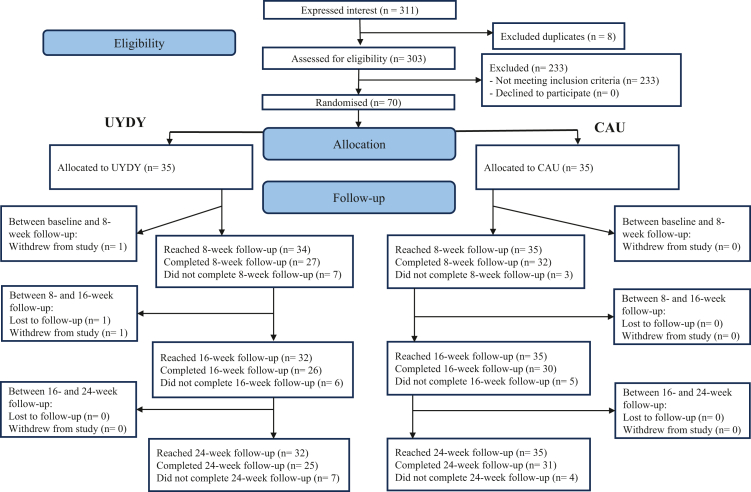
The Consolidation of Standards for Reporting Trials (CONSORT) Diagram, showing the number of participants at each point of the study. Note: Lost to follow-up: Participant stopped responding to questionnaire prompts for this and all subsequent questionnaires, but did not request to be withdrawn from study. UYDY: Understanding You, Discovering You intervention. CAU: Care as usual.

A total of 303 individuals expressed interest in participation. A total of 70 eligible participants consented to be randomised, with half randomised to each arm of the study. Of these 70 participants, 40 nominated a carer to participate. Of these 40 carers, 30 consented, 10 in UYDY and 20 in CAU. See Fig. 1 for CONSORT diagram and Supplementary Table S4 for exclusion reasons.

See Table 1 for demographic information about participants at baseline. Most participants were white, female, and students. The UYDY arm had a higher percentage of individuals who were currently taking medications for mental health (*n* = 18, 51%), compared to the CAU arm (*n* = 10, 29%).

**Table 1:** Baseline Characteristics of the Understanding You, Discovering You (UYDY) intervention and the care as usual (CAU) groups.

Variable	UYDY (n randomised = 35), mean (SD), n (%) as appropriate	CAU (n randomised = 35), mean (SD), n (%) as appropriate
Stratification Variable: Age		
16–17	12 (34%)	13 (37%)
18–25	23 (66%)	22 (63%)
Overall sample: Age	19.60 (2.69)	20.00 (3.36)
Sex Assigned at Birth		
Male	5 (14%)	11 (31%)
Female	30 (86%)	24 (69%)
Gender		
Male	6 (17%)	10 (29%)
Female	26 (74%)	22 (63%)
Nonbinary	2 (6%)	2 (6%)
Other	1 (3%)	1 (3%)
Ethnicity		
White	34 (97%)	29 (83%)
Mixed/Multiple Ethnic Groups	0 (0%)	2 (6%)
Asian/Asian British	0 (0%)	1 (3%)
Black/African/Caribbean/Black British	1 (3%)	2 (6%)
Other	0 (0%)	1 (3%)
Education		
None	4 (11%)	2 (6%)
GCSE (or equivalent)^a^	9 (26%)	15 (43%)
A Level (or equivalent)^b^	16 (46%)	6 (17%)
NVQ/BTEC/Vocational degree (or equivalent)^c^	0 (0%)	1 (3%)
Undergraduate degree (e.g. BSc, BA)	4 (11%)	8 (23%)
Postgraduate degree (e.g. MSc, MA)	2 (6%)	3 (9%)
Employment status^d^		
Employed full-time	4 (11%)	6 (17%)
Employed part-time	9 (26%)	6 (17%)
Unemployed	4 (11%)	5 (14%)
Student	25 (71%)	24 (69%)
Currently taking medication for mental health (%)	18 (51%)	10 (29%)

a GCSE stands for the General Certificate of Secondary Education usually taken at around the age of 16 in the United Kingdom (UK).

b A Levels stand for Advanced Levels, a two-year qualification in the UK.

c NVQ stands for National Vocational Qualifications while BTEC stands for the Business and Technology Educational Council, a range of vocational qualifications in the UK.

d Participants could endorse more than one category.

Autistic young people completed questionnaires at baseline and at 8-, 16-, and 24-week follow-up, and carers completed questionnaires at baseline and 16-week follow-up. Table 2 provides information at each time point about data completeness, calculated as percentages of the number of participants in each arm who completed follow-up measures at each timepoint. There was greater attrition in the UYDY arm compared to CAU. One individual in UYDY withdrew from study procedures without providing a reason, and no participants withdrew from CAU. One individual withdrew from the UYDY intervention after randomisation but before sessions began due to their work schedule.

**Table 2:** Data completeness of young person participants and carer participants of the Understanding You, Discovering You (UYDY) intervention and the care as usual (CAU) groups.

	UYDY young people (n randomised = 35)			CAU young people (n randomised = 35)		
Week	8	16	24	8	16	24
Warwick Edinburgh Wellbeing Scale	29 (83%)	26 (74%)	26 (74%)	33 (94%)	32 (91%)	31 (89%)
Autism Social Identification	29 (83%)	26 (74%)	26 (74%)	33 (94%)	32 (91%)	31 (89%)
EQ-5D-5L	29 (83%)	26 (74%)	26 (74%)	33 (94%)	32 (91%)	31 (89%)
Interpersonal Support Evaluation List—Short	28 (80%)	26 (74%)	26 (74%)	32 (91%)	31 (89%)	31 (89%)
UCLA Loneliness Scale	28 (80%)	26 (74%)	26 (74%)	32 (91%)	31 (89%)	31 (89%)
Adapted Client Service Receipt Inventory	27 (77%)	26 (74%)	25 (71%)	32 (91%)	30 (86%)	31 (89%)

	UYDY carers (n consented = 10)	CAU carers (n consented = 20)
Week	16	16
ASCOT-CARER	8 (80%)	15 (75%)
Warwick Edinburgh Wellbeing Scale	7 (70%)	15 (75%)

A summary of CAU accessed by all participants is found in Table 3. Across both arms, participants were most likely to utilise medications and primary and community care.

**Table 3:** Health and social care of the Understanding You, Discovering You (UYDY) intervention and the care as usual (CAU) groups at different timepoints.

	Baseline		8-Week follow-up		16-Week follow-up		24-Week follow-up	
	UYDY	CAU	UYDY	CAU	UYDY	CAU	UYDY	CAU
Inpatient^a^	2 (6%)	1 (3%)	3 (9%)	1 (3%)	3 (9%)	2 (6%)	2 (6%)	0 (0%)
Outpatient^a^	8 (23%)	6 (17%)	10 (29%)	10 (29%)	5 (14%)	3 (9%)	5 (14%)	8 (23%)
Community-based day services	9 (26%)	8 (23%)	15 (43%)	8 (23%)	9 (26%)	9 (26%)	8 (23%)	9 (26%)
Primary and community care contacts	16 (46%)	14 (40%)	13 (37%)	15 (43%)	10 (29%)	12 (34%)	13 (37%)	11 (31%)
Criminal justice	1 (3%)	0 (0%)	1 (3%)	0 (0%)	0 (0%)	0 (0%)	0 (0%)	0 (0%)
Medication	22 (63%)	15 (43%)	16 (46%)	13 (37%)	20 (57%)	14 (40%)	20 (57%)	14 (40%)

N = 35 for UYDY at each time point; N = 35 for CAU at each time point.

a Mental and Physical Healthcare.

UYDY groups were delivered to five groups of participants, with group sizes ranging from five to nine. Group facilitators completed session-by-session fidelity checklists (*N* = 30 sessions) indicating completion of group content and objectives. Facilitators completed almost all checklists (*n* = 29, 97%). They reported covering all content and objectives across all sessions, with the exception of one rater who noted that an objective in Session 1 of one cohort was “partly” met.

In terms of intervention acceptability, 77% (*n* = 27) of participants allocated to UYDY attended at least three group sessions. Participants completed a median of 5 sessions (IQR [3–6]).

The two arms had a comparable number of adverse events (UYDY: 6, CAU: 7), though participants in the UYDY arm were more likely to report experiencing a serious adverse event, with two events (unrelated to study participation) reported in this arm by one participant compared to none in CAU.

**Fig. 2:**
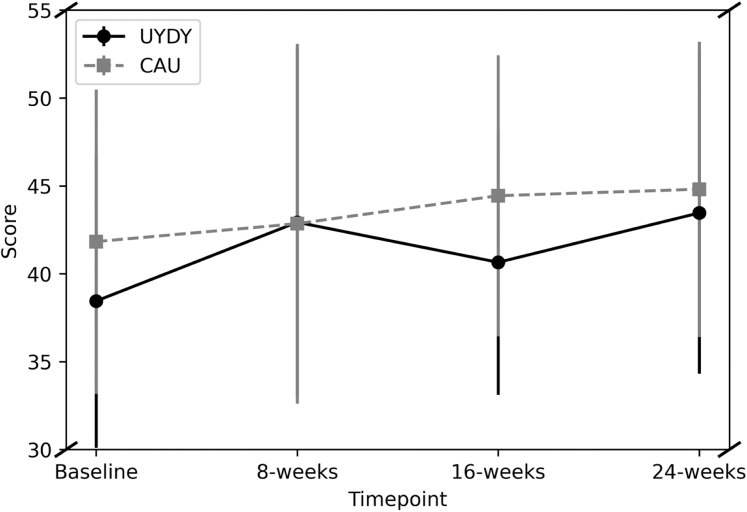
Line graph of Warwick Edinburgh Wellbeing Scale (WEMWBS)^1^ measure mean and standard deviation (SD) by timepoint and group (Understanding You, Discovering You [UYDU], and care as usual [CAU]). ^1^ Minimum score: 14, Maximum: 70, y-axis truncated. Standard deviation presented to illustrate variance of measures rather than precision of estimated mean.

Descriptive statistics including outcome measure variances for young person participants are shown in Table 4 and for carer participants in Supplementary Table S5. See Fig. 2 for a graphical depiction of change in WEMWBS across timepoints by group. The study was powered for feasibility and not effectiveness aims, so we did not conduct inferential statistics. From visual inspection, participants allocated to CAU had higher baseline wellbeing scores, and there was a different pattern of change in wellbeing scores at each follow-up timepoint by group.

**Table 4:** Outcome measures for young person participants in the Understanding You, Discovering You (UYDY) intervention and the care as usual (CAU) groups.

Variable	UYDY young people (n randomised = 35), mean (SD), n (%) as appropriate					CAU young people (n randomised = 35), mean (SD), n (%) as appropriate				
	Baseline (n = 35)^a^	8-week follow-up (n = 29)^a^	16-week follow-up (n = 26)^a^	24-week follow-up (n = 26)^a^	Change (baseline to 16 weeks)	Baseline (n = 35)^a^	8-week follow-up (n = 33)^a^	16-week follow-up (n = 32)^a^	24-week follow-up (n = 31)^a^	Change (baseline to 16 weeks)
Wellbeing (WEMWBS)										
M (SD)	38.45 (8.35)	42.93 (9.87)	40.65 (7.53)	43.46 (9.14)	3.04 (6.40)	41.83 (8.66)	42.85 (10.23)	44.44 (8.00)	44.81 (8.40)	2.66 (7.15)
Autism social identity										
Total; M (SD)	4.62 (0.80)	4.83 (0.76)	4.68 (0.61)	4.80 (0.73)	0.09 (0.47)	4.62 (0.88)	4.64 (1.05)	4.35 (0.98)	4.61 (1.01)	−0.08 (0.58)
Quality of life (EQ-5D-5L)										
M (SD)	0.69 (0.19)	0.73 (0.22)	0.73 (0.21)	0.76 (0.21)	0.04 (0.19)	0.77 (0.15)	0.77 (0.16)	0.81 (0.19)	0.80 (0.16)	0.03 (0.15)
Inter-personal support										
Appraisal; M (SD)	10.74 (2.89)	10.64 (2.60)	11.00 (2.86)	11.31 (2.15)	0.12 (2.86)	11.97 (2.49)	12.06 (2.84)	11.94 (2.53)	11.74 (2.74)	−0.10 (2.07)
Belonging; M (SD)	8.43 (3.12)	9.11 (2.30)	9.19 (1.86)	9.54 (2.21)	0.58 (3.02)	8.60 (3.09)	9.41 (2.89)	9.32 (2.59)	9.16 (2.70)	1.06 (2.11)
Tangible; M (SD)	12.09 (3.11)	12.21 (2.59)	11.96 (2.43)	12.35 (2.21)	−0.38 (2.80)	12.57 (2.54)	12.19 (2.57)	12.13 (2.51)	12.03 (2.24)	−0.35 (2.51)
UCLA loneliness scale										
M (SD)	9.20 (2.17)	8.86 (2.00)	9.19 (2.00)	8.69 (1.81)	0.00 (1.23)	8.49 (2.38)	8.06 (2.36)	8.03 (2.52)	8.10 (2.51)	−0.39 (1.71)
Health and social care										
Accessed care in last 3 months (%)	30 (86%)	21 (60%)	21 (60%)	20 (57%)	–	26 (74%)	23 (66%)	20 (57%)	21 (60%)	–

a Number of participants who completed WEMWBS outcome measure at that timepoint.

The qualitative analysis resulted in five themes (see Supplementary Table S6), described below.

### Finding out about the study

Participants often discovered the study passively online through autism charities or social media. Many participants were not actively seeking out research or support, but heard about the study and became interested. To recruit a representative sample, some participants recommended advertising in spaces focused on autistic individuals, though others also recommended advertising in general settings, such as educational settings, workplaces or social media sites including TikTok and YouTube. One participant (UYDY016) described how they had found some other research opportunities through TikTok, “which is quite nice, because they tend to be videos and it explains [the study] very thoroughly, like what is happening.”

### Understanding of the study

Participants gave largely positive feedback regarding the information they were provided about the study. Participants noted that it was understandable, written in an accessible way, and had a good amount of information that was well designed and formatted. Some participants stated that there was either too much information leading to a struggle to digest the information, or too little information, which led to a lack of clarity. Participants understood the randomisation process, though some carers of participants were unaware of randomisation or that the UYDY groups were offered to those in CAU upon completion of their final follow-up.

### Goals and preferences for the study

Many participants had goals for the study/group that motivated their participation, such as improving wellbeing. Other participants had no specific goals. Several participants mentioned their dissatisfaction with CAU and their perception that UYDY would be a preferable alternative. Of those who had preferences about their allocation, most preferred UYDY allocation because they wanted additional support, whereas the CAU arm was described as “just kind of normal, like there's nothing really different other than doing the questionnaires” (CAU015). Participants varied in terms of the strength of their preferences, with many of them not holding strong preferences, partially driven by the waitlist incentive.

### Participating in study tasks

Many participants felt that being interested in research facilitated their engagement with the study. They described other facilitators of study engagement like good communication and support from study staff, as well as financial incentives and reminders of study tasks. Some participants noted challenges of trial participation in terms of the number of study tasks. One challenge was that participants allocated to CAU sometimes felt that they did not have much to do, making the trial less engaging compared to those in the UYDY arm. Conversely, some participants noted that the time commitment was too much, even in the CAU arm.

Participants found the questionnaires to be acceptable and easy to complete independently. Several participants believed the questionnaires captured their opinions about their wellbeing and did not miss any important domains. Conversely, some participants noted challenges including ambiguous wording of questions and difficulty translating their experiences into the questionnaire response options. Some felt that the questionnaires were too long and repetitive, which acted as a barrier for engagement and completion. Another limitation of the questionnaires was the poor interface for completion via mobile phone, the preferred method of many participants. To mitigate some of the challenges, participants offered suggestions, such as providing more support in the questionnaire process or providing free text response options.

### Reflecting on study outcomes

Participants reported a positive experience of the research, and an investment in study outcomes. Many participants noted that the study and groups were well-run in terms of their organisation and the attitudes of the study team, and that they enjoyed the routine of participation. Despite general positive feedback, when asked about changes in one's life or wellbeing during or after the study, participants were more likely to discuss negative changes. For example, one participant (UYDY031) noted that their mental health “dipped quite a bit” during the study. Participants with poorer outcomes generally attributed these outcomes to external circumstances rather than participation in the study, indicating the range of stressors that autistic young adults experience and the need for support.

## Discussion

Our co-produced feasibility RCT indicated that a full-scale trial should be feasible since trial procedures were seen as acceptable, but further work to strengthen the design would be needed first. In summary, qualitative evidence indicated that the intervention was acceptable to participants, as did quantitative evidence of attendance rates: 77% (*n* = 27) of participants allocated to UYDY attended at least three of six sessions. We met our recruitment targets on time, and retention at 16-weeks was acceptable, although a significant limitation is that fewer participants completed outcome measures in UYDY compared to CAU. Participants found the outcome measures relevant including the primary WEMWBS wellbeing measure. In terms of CAU, participants most often accessed medication, primary and community care.

A key goal of a feasibility study is to highlight areas for improvement and attention in any future full trial. To that end, we identified several areas to strengthen before proceeding to full trial. Importantly, we identified that there was a higher rate of attrition in those allocated to UYDY compared to CAU. It is possible that both attending the groups and completing measures was considered too much burden by participants allocated to UYDY. While some participants mentioned this, they were in the minority. Alternatively, we know that the landscape of post-diagnostic support for autistic adults in the UK is highly variable.^29^ Those allocated to CAU may therefore have felt obliged to remain engaged in the study in order to access the UYDY groups after study participation ended, due to a lack of available services in their region. However, outcome completion was not mandated to later access UYDY, and not all participants allocated to CAU opted to attend the groups: this was taken up by 43% (*n* = 15) of CAU participants.

In contrast, carer take-up of the study was lower in CAU compared to UYDY. It is possible that both autistic young people and their parents/carers felt ambivalent about carer participation in a study focused on a successful transition to adulthood, which for many individuals means increased independence from family support. In a full trial, measures such as increased incentive payments and videos from autistic young people explaining the importance of follow-up completions may improve retention rates. Importantly, any effectiveness trial would need an internal pilot to ensure that these issues were addressed before being able to proceed to full trial.

Participants allocated to UYDY had lower baseline wellbeing scores than participants in CAU. While there was a comparable number of adverse events across groups, there were two serious (unrelated) adverse events in one participant allocated to UYDY compared to zero in CAU. Autistic individuals are at greater risk of suicide and self-harm,^3^ and the transition to adulthood presents a challenging time for risk management given the changing context^9^ in terms of support services, frequency of moving localities, and higher rates of emotional dysregulation. Robust risk management standardised operating procedures are needed in clinical trials with autistic people, and especially those in this age group, as was the case in this study.

In terms of intervention acceptability, there was evidence from this study that the intervention was acceptable in terms of the high proportion of participants who attended at least half of the sessions. Further, around one quarter of participants allocated to UYDY attended two or fewer appointments: this is a limitation that should be addressed in future work by investigating the reasons for not attending sessions and the impact of number of sessions attended.

A full trial is required to understand whether the intervention is effective in improving wellbeing in autistic young people. While we cannot evaluate effectiveness from the current study, it should be noted that there was a substantial difference in baseline wellbeing scores across groups, and there were not large changes in wellbeing in the UYDY group compared to CAU at 16-weeks. In the qualitative work, when asked about changes in wellbeing, participants most often discussed negative changes that had occurred in their lives during participation in the trial. However, these were linked to broader life events, and in the context of the UYDY group reporting lower rates of wellbeing and higher rates of mental health medication use at baseline. Qualitative feedback about UYDY was largely positive. The current study does provide information to inform the decision about whether to proceed to a full trial to test effectiveness, as well as economic costs and benefits of the intervention. The decision about whether a full trial is warranted reflects a number of considerations including: (i) if it is routinely delivered in some parts of the healthcare system and therefore needs to be tested; and (ii) if there is sufficient proof-of-concept for the intervention mechanism to suggest it has promise. Groups with similar features to UYDY are routinely offered in some parts of the NHS in the UK,^29^ and there is some evidence supporting target mechanisms like positive autism identity.^30^

Qualitative findings also provided useful insights as to how to design appealing trials for autistic young people. These will be used in designing any full trial of the UYDY groups, and could be applied in other trials, including those with wider age ranges than the current study. Some key ideas included: using videos on social media and targeting general, non-autism groups for recruitment; co-producing study information such as participant information leaflets to support participation in the study and understanding of processes such as randomisation; incentives for questionnaires being crucial to motivation to complete these; that outcome measures with ambiguous wording should be avoided or support offered for their completion; and that the team's efforts to engage in clear and well organised communications led to a positive experience of the study and high engagement and investment. Importantly, participants found randomisation procedures acceptable. The level of acceptability was likely increased by the fact that the UYDY groups were offered to those in CAU after their participation in the study ended. At the same time, a proportion of those allocated to CAU did not take up groups, which may be reflective of the rapidly changing life circumstances for this group, since participants did generally express a preference to be allocated to UYDY. In the qualitative study, when discussing study outcomes, participants were particularly likely to recall negative life events and how these had impacted on wellbeing during the study timeframe. In a full trial, more work should be done to longitudinally understand the effect of participating in the trial as well as UYDY groups.

We should emphasise that our findings are limited by the sample not being representative of the autistic population, especially in terms of gender and ethnicity. Significant efforts were made during the feasibility trial to increase diversity, for example, contacting more specific community groups in geographical areas with higher ethnic diversity, but these were not ultimately successful. We identified that we recruited a more representative sample through parent groups than through national autism charities. A full trial must recruit a more representative sample of participants. Potential strategies include using national networks to recruit from parent and community groups, approaching schools/colleges in areas with higher rates of ethnic and socioeconomic diversity, and a targeted social media campaign. Additionally, a dedicated diversity strategy group could benchmark progress in terms of recruitment diversity and modify the recruitment strategy where necessary. Representativeness of the sample is a significant limitation of the current work, and one that future studies must address. Moreover, because of the scale of this feasibility trial, there were differences in demographics between groups (e.g. sex, gender, ethnicity, mental health, education level) which were likely exacerbated by the sample not being sufficiently diverse, as well as differential attrition in UYDY and CAU. These are significant concerns for any future trials with a similar design, and comprehensive co-production work, plus a pilot phase, would be needed if a full trial was undertaken, to ensure that participants from less represented groups were willing to participate.

In conclusion, a full trial of the UYDY groups is likely to be feasible because we were able to recruit and retain participants, and they reported that they found the trial procedures and interventions to be acceptable. Adaptations to the protocol will be required to address differential attrition across trial arms and to recruit a more representative sample. Autistic young people described feeling motivated to participate in the research. Participants appreciated the use of co-production, including of the intervention, and study information and procedures. Such elements will be essential in any future trials with this population.

## Contributors

KC, LA, BB, LC, LP, BR, AR, and WM conceptualised the study, acquired funding and oversaw the management and conduct of the study. KC, AB, BR, and WM further trained and supervised the group facilitators. RAV and AB led on trial administration and participant facing processes. AB and BR led on participant recruitment. RAV and AB were responsible for curating and cleaning the quantitative data. KC, RAV, and BR wrote the original draft of the manuscript. RAV conducted the quantitative analysis and prepared all tables and figures. Trial data were accessed and verified by the lead statistician MVM. RAV and MVM accessed and verified the underlying study data. The qualitative data were collected by KC, RAV, AB, LA, LI, and BR. Qualitative data analysis was conducted by KC, RAV, LA, LI, and BR. All authors reviewed and edited the draft of the manuscript. All members of the trial management group had full access to all the data in the study and accept responsibility for the decision to submit for publication.

## Data sharing statement

Participants were asked whether they consented to their anonymised data being shared with other researchers who have received appropriate ethical approval for their research. This will be made available to other eligible researchers on request following publication, with investigator support and a data access agreement.

## Declaration of interests

Since the start of the study, WM became a Trustee of Autistica, which is an unpaid role. All remaining authors declare no competing interests.

## Appendix A Supplementary data

Supplementary Protocol
