# Supplementary material for: Assessing the feasibility of a co-produced peer-group intervention for supporting wellbeing during the transition to adulthood among autistic 16–25-year-olds (ATAG): a randomised controlled feasibility trial
Source: eClinicalMedicine. 2026 Apr 7;94:103859. doi: 10.1016/j.eclinm.2026.103859 (PMC13090626; doi:10.1016/j.eclinm.2026.103859)
Supplement: Supplementary Protocol [file mmc1.pdf]

Autism Transition to Adulthood Groups (ATAG): Protocol for a feasibility RCT of a new peer-group intervention to promote successful transition to adulthood for autistic young people

Kate Cooper[1,2], Annabel Burnley[2], Leon Allain[2,3], Bryony Beresford[4,5], Laura Crane[6,7], Maximiliano Vazquez Morales[8,9], Lucy Portway[2,3], Benjamin Redmayne[3], Ailsa Russell[2], William Mandy[1].

1. Research Department of Clinical, Educational, & Health Psychology, University College London, London, United Kingdom.
2. Centre for Applied Autism Research, Department of Psychology, University of Bath, United Kingdom.
3. Ambitious about Autism, London, United Kingdom.
4. School for Business and Society, University of York, United Kingdom.
5. Social Policy Research Unit, University of York, United Kingdom.
6. Centre for Research in Autism and Education (CRAE), IOE, UCL's Faculty of Education and Society, University College London, London, UK
7. Autism Centre for Education and Research (ACER), Department of Disability, Inclusion and Special Needs, School of Education, College of Social Sciences, University of Birmingham, UK
8. Population Health Sciences, University of Bristol, Bristol, UK
9. Bristol Trials Centre, University of Bristol, Bristol, UK

Corresponding Author: Kate Cooper, k.r.cooper@ucl.ac.uk

Acknowledgements: Ambitious about Autism and Youth Advisors from the Ambitious Youth Network, Alison Worseley, members of the reference group for UYDY, ATAG Trial Steering Committee

Keywords: Autism; Transition to Adult Care; Peer Group; Post-Diagnostic Support; Internet-Based Intervention

Word count: 4646

Abstract

Introduction

Autism is a lifelong neurodevelopmental condition diagnosed on the basis of differences in social communication, interaction, and repetitive behaviours, including sensory sensitivities. Autistic individuals without intellectual disabilities often face barriers to positive adult outcomes and are at high risk for poor health, including mental health issues, which could be mitigated by improving well-being. Young people should receive support to increase their well-being during the transition to adulthood, when social and family support often reduces. This is the protocol for a feasibility randomised controlled trial (RCT) of an online peer-group intervention, "Understanding You, Discovering You" (UYDY). Objectives included assessing recruitment and retention rates, acceptability of procedures, characterising usual care, assessing the acceptability of UYDY and CAU, and calculating outcome measure variances for a full trial.

Methods and analysis

This 2-arm parallel feasibility RCT includes a nested qualitative evaluation. Seventy participants aged 16-25 years old with a clinical autism diagnosis will be randomised on a 1:1 basis to UYDY or Care as Usual (CAU). Exclusion criteria include risk of harm to self or others, receipt of post-diagnostic support in the past 12 months, and literacy levels such that the written session materials are not accessible. UYDY, a six-week online peer-group intervention, will cover topics such as understanding autism, problem-solving, and accessing services, and is facilitated by an autistic person and social care professionals. The main outcomes from the feasibility trial will be collecting data on: (1) recruitment and retention rates; (2) the acceptability of randomisation and outcome measurement procedures; (3) CAU accessed by participants; (4) acceptability of the interventions; (5) clinical outcome measure variances (see below). See the statistical analysis section below for how this will be assessed in the current study.Clinical outcomes will be measured at baseline, and 8-, 16-, and 24 weeks post-randomisation. The primary clinical outcome is well-being, assessed using the Warwick Edinburgh Mental Wellbeing Scale (WEMWBS). Secondary clinical outcomes include autism social identification, quality of life, social support, and loneliness. Adverse events will be monitored and reported. Carer impact will also be measured. Participants will be recruited from England and Wales via charities and NHS services. Qualitative interviews will be conducted to explore the acceptability of trial participation including randomisation and the interventions.

Ethics and dissemination

Ethical approval has been obtained from the HRA and NHS REC (23/WA/0113). Informed consent will be collected from all participants (see Supplemental Material for an example consent form). Results will inform the design of a full RCT and will be disseminated through peer-reviewed journals, conferences, and stakeholder events.

Strengths and limitations of this study

- Feasibility randomised controlled trial of a new intervention for autistic 16–25-year-olds
- Co-produced intervention and trial design
- Nested qualitative evaluation will investigate lived experiences of the trial and interventions
- It will not be possible to make claims about the effectiveness of the intervention from this feasibility study

Trial registration: ISRCTN10513626.

Introduction

Autism is a lifelong neurodevelopmental condition diagnosed on the basis of differences in social communication and interaction and restricted and repetitive behaviours, including sensory sensitivities[1]. In this paper we focus on autistic young people without intellectual disability. Autistic individuals experience many barriers to good adult outcomes, and are at risk of poor health[2] and social outcomes[3]. This includes being at high risk for mental health problems[4], which are more common in those aged 16-24 years as compared to those aged 0-15 years[5]. It is important to research ways to reduce risk factors at this life stage. Moreover, improving well-being has been shown to reduce severity of mental health problems and prevent their onset [6,7] so well-being is a promising treatment target in all groups, and especially populations at increased risk for mental health problems.

For all young people the transition to adulthood is a moment of opportunity but also one of risk, when existing supports and structures are lost at a time of increasingly complex demands. In this paper we define the transition to adulthood as applying to young people aged 16-25 years, which is in line with service provision for young adults in the UK, however, there is debate about the definition of adolescence, youth and young adulthood, and the boundaries between these life stages in both the general population[8] and autistic individuals specifically[9]. This life stage sees a reduction of formal and informal support (e.g., in education, healthcare, child services, family), without a corresponding increase in support from adult services[10]. Many report being ill-prepared for this transition, during which expectations about their ability to live independently might outweigh their capabilities[11], and with uncertainties about where support can be accessed since the move beyond the family and school environments[12].

It is crucial that autistic individuals receive evidence-based support to improve well-being, and this should be tailored to their needs as autistic individuals transitioning to adulthood. At present, such support is lacking for autistic people of all ages. The 2021 Westminster Commission on Autism report surveyed 585 autistic people and their families in the UK and found that the time following an autism diagnosis was stressful for 78% of respondents and

that more tailored information about autism and access to support services would be welcomed, alongside peer-support[13]. A study about the provision of support to autistic adults without intellectual disabilities identified high variation in services across the country. Post-diagnostic support frequently included support to increase knowledge and understanding of autism, support to develop problem solving skills and information about services and support. In a UK observational study, autistic individuals who received post-diagnostic support had improved General Health Questionnaire scores, and improved daily living outcomes, whereas those who received a diagnosis without support did not see such improvements[14]. Qualitative evidence and feasibility and pilot studies provide support for the potential of such interventions in young people. Small feasibility studies of internet-delivered autism support for young autistic adults in Sweden showed promise in terms of intervention attendance, acceptability of the intervention and increase in autism knowledge[15]. A small-scale RCT of a group-based autism post diagnostic support programme for autistic young adults in Canada demonstrated improvements in quality of life[16]. A qualitative evaluation of an autistic-led autism support programme in the UK found positive responses from autistic participants when it was delivered both in person and online[17][18]. A feasibility study of peer-based support for autistic adolescents and adults in the USA demonstrated promise in terms of participant satisfaction ratings[19]. Further, a recent systematic review identified that telehealth services show promise in terms of clinical effectiveness and economic impact[20], providing support for online intervention delivery.

In adults, such support to understand autism, develop problem-solving skills and increase access to relevant services is typically delivered immediately following diagnosis. However, we know little about support offered to young people, and it may be that such support is required years after a diagnosis. This is particularly likely in cases when the diagnosis was provided at an age when the young person was not able to understand it, or at a time when they were not willing or able to engage with the information. In this paper, we therefore define post-diagnostic support as interventions which are offered at any point after a diagnosis of autism which aim to help an individual learn about autism, develop strategies to manage daily life and make sense of and accept their autism diagnosis, irrespective of timing with regard to the autism assessment and diagnosis. This definition also fits with adolescent and young adult development, which marks an important period in the negotiation of identity and social relationships[21][22]. Receiving a diagnosis of autism has significant implications for this process[23]. There is evidence that having a positive sense of autism identity, autism self-acceptance, and autism community connectedness is associated with better well-being and mental health outcomes when measured quantitatively[24]. Therefore, autism related support which aims to modify these factors may succeed in

improving well-being, mental health and quality of life, and this may be particularly effective when delivered to those who are transitioning to adulthood.

There have been growing calls for more community-led and co-produced supports and services, and for greater autistic involvement in research and practice[25]. Post-diagnostic support which is co-designed by members of the autistic community is therefore more likely to be perceived as credible and acceptable to those who will be accessing it.

The above evidence suggests the potential of a co-designed online group intervention which promotes autism-related knowledge, social connection and social support within the autistic community. We have co-designed an intervention called *Understanding You, Discovering You* with the autistic community. To develop evidence-based interventions, a randomised controlled trial (RCT) is needed, and the first step is to understand the feasibility of running such an RCT.

We are therefore conducting a feasibility randomised controlled trial where participants will be randomly allocated eligible and consenting participants at a 1:1 ratio to either Understanding You, Discovering You (UYDY) groups and Care as Usual (CAU). A feasibility design has been selected because this will be the first trial of its kind, testing a new co-produced intervention for autistic young people.

Our objectives were as follows:

1. Assess the rates of recruitment and retention to inform the design of a full-scale RCT
2. Assess the acceptability of randomisation and outcome measurement procedures to participants
3. Characterise Care As Usual (CAU)
4. Assess the acceptability of the UYDY intervention and CAU
5. Calculate outcome measure variances for use in a power calculation for a full trial

Methods and analysis

Design

This is the protocol for a 2-arm parallel feasibility RCT with a nested qualitative evaluation. We follow here the SPIRIT reporting guidelines (see table 2)[26].

Study Setting

This trial will be delivered through a partnership between academic institutions, charities (primarily Ambitious and Autism), and autistic individuals and other stakeholders, including health and social care professionals. Recruitment will be conducted via charities, e.g.

Ambitious about Autism, National Autistic Society and Autistica, and lists of potential research participants e.g. at the Centre for Applied Autism Research at the University of Bath. We will also recruit from one pilot NHS autism service as a participant identification centre (PIC) to understand the feasibility of recruitment in NHS settings.

Eligibility criteria

Inclusion criteria

• Aged 16-25 years at the point of randomisation

• A clinical diagnosis of Autism Spectrum Disorder verified by clinical letter or report.

Exclusion criteria

• Participants who report that they have received any autism post-diagnostic support over the past 12 months will be excluded from the study. This is operationalised as any specific support from a health or social care professional about what an autism diagnosis means, how autism may affect you, and how to find support as an autistic individual. This includes meeting with a professional in an individual or group setting to talk about what autism is. It does not include any self-help through websites or reading, nor does it include support from educational professionals.

• Risk of harm to self that would mean that this intervention is not clinically appropriate, assessed using item 9 of the PHQ-9 and follow up by a clinician. Where an individual requires intensive mental health support to reduce their level of risk, the individual will not be eligible for this study.

• Risk of harm to others such that group participation would be contraindicated. This will be assessed by asking a series of questions about the individual’s school exclusion and forensic history.

• English, non-English & Welsh literacy levels such that the intervention materials are inaccessible.

Interventions

Understanding You, Discovering You

The intervention is the Understanding You, Discovering You (UYDY) groups, which is a peer-group intervention aimed at supporting the transition to adulthood in autistic young people. This multi-component intervention aims to: (i) provide education about autism (ii) enhance capacity to identify and make use of relevant health and social care services when required, and (iii) generate affiliation to and social connections within the autistic community.

A process of co-design led by Ambitious about Autism generated a six-week online peer-group intervention. The process of co-design is documented in depth in a separate paper[27].

Six online, hour-long, weekly sessions, in groups of 7-10 participants, will be co-facilitated by an autistic person and two professionals from a range of social care and educational backgrounds, e.g., social workers, occupational therapists, experienced support workers. The facilitators lead the online sessions, support participation of discussions, and take responsibility for the welfare of participants during the sessions. They also facilitate after-session debriefs, complete fidelity forms and attend regular supervisions. The autistic facilitators also role model a positive autistic identity and provide positive affirmation to young people's experience of autism.

Facilitators will receive monthly supervision of their practice and seven hours training provided by two qualified Clinical Psychologists and the Ambitious about Autism programme manager (KC, WM & BR) which will cover: understanding the needs of autistic young people; current service provision for this group and signposting; adapting communication for autistic young people; techniques in delivering online group interventions.

Following each group session, one facilitator will complete a Facilitator Record Form (FRF) which will record which elements of the intervention were completed and any issues identified with the UYDY intervention including observations of participant engagement.

The six UYDY sessions are structured using a facilitator manual and standardised session materials in the format of presentation slides and private online workspaces where participants can share answers to questions and participate in quizzes. The sessions cover topics through didactic and experiential learning. Young people are encouraged to share their experiences and knowledge linked to the topic of each session in structures, and the autistic facilitator also is prompted to share their experiences to provide examples. Topics covered over the six weeks include: understanding the autism label; autistic strengths; problem solving and goal setting; disclosing one's diagnosis and understanding one's rights; identifying needs and accessing services when appropriate; identifying and meeting one's social needs. Participants receive the agenda and session materials for each session by email.

Educational resources will be offered to those nominated by the participant as a supporting person, whether a carer, partner, or family member. These are written resources which map onto the session content delivered to young people. They are sent to carers at the start of the study, and carers are encouraged to read these week-by-week as the young person attends the group sessions.

Participants receiving UYDY will also be able to access care as usual, which will be measured for participants in both arms of the trial.

The UYDY Programme Team will contact any individuals who do not attend, to encourage them to attend future sessions.

Care as usual (CAU)

Since this is a pragmatic trial, CAU will continue without restriction, including referrals to health and social care provision for autistic young people. Participants allocated to CAU will be given the opportunity to access the UYDY groups upon completion of their final follow-up.

Table 1

Quantitative trial assessments and key participant-related procedure

**Data collection timepoint (→)**	**Pre-randomisation**		**Post-randomisation**			
**Key quantitative measures and trial procedures for autistic young people**	**Identification / Screening**	**Baseline**	**1 – 8 weeks**	**8-weeks**	**16-weeks**	**24-weeks**
Screening	●					
Eligibility assessment	●	●				
Consent to join trial		●				
Receive Understanding You, Discovering You (UYDY) or Care as Usual (CAU)			Access UYDY or CAU			
UYDY facilitator records forms			●			
Warwick Edinburgh Mental Wellbeing Scale *(WEMWBS - Primary Outcome at 16-weeks)*		●		●	●	●
Autism social identification measure		●		●	●	●
Quality of life / utility (EQ-5D-5L)		●		●	●	●
Interpersonal Support Evaluation List - short		●		●	●	●
UCLA loneliness scale		●		●	●	●
Health and social care resource use (Client Service Receipt Inventory)		●		●	●	●
**Key quantitative measures and trial procedures for carers**						
Consent to join trial and complete outcome measures	◊	◊				
Receive carer educational resources			Access carer educational resources			
Social-care related quality of life for carers (ASCOT-carer)		◊			◊	
Warwick Edinburgh Mental Wellbeing Scale *(WEMWBS)*		◊			◊	

***Key**: ● data capture / outcome measures; completion methods may vary depending on participant preferences. ◊ completed by caregiv*

OutcomesThe main outcomes from the feasibility trial will be collecting data on: (1) recruitment and retention rates; (2) the acceptability of randomisation and outcome measurement procedures; (3) CAU accessed by participants; (4) acceptability of the interventions; (5) clinical outcome measure variances (see below). See the statistical analysis section below for how this will be assessed in the current study.

The primary clinical outcome is the self-report outcome measure Warwick Edinburgh Wellbeing Scale (WEMWBS) at 16-weeks post-randomisation. Well-being was the measure which best captured the overall intended outcomes of the multifaceted intervention. This measure is validated for use in this age group and with good internal consistency in autistic adults[28],[29]. This 14-item scale was found to have good internal reliability in a sample of autistic young people aged 15-22 years, and we will assess internal reliability of the measure in 16–25-year-olds in this study. Items include “I've been feeling confident”, scored on a Likert scale from 1 “none of the time” to 5 “all the time”, with higher scores indicating better wellbeing.

The secondary clinical outcome measures are as follows. The autism social identification measure[30],[31] is a measure of affiliation with the autistic community and identity, with items such as “I am glad to be autistic”. This 14-item scale has good reliability and construct validity in autistic adults[32] and good reliability in autistic young people aged 15-22 years. The EQ-5D-5L will be used to measure quality of life and has been used in previous work with autistic adults[14,33]. The Interpersonal Support Evaluation List - short[34] will be used to measure perceived social support, as previously used with autistic adults[14]. The four-item short UCLA loneliness scale (ULS-4)[35] will be used to measure experiences of distress due to a discrepancy between actual and desired social connection.

A use of services questionnaire based on the Client Service Receipt Inventory[36] will be used to characterise CAU by thorough measurement of the additional health and social care services accessed by all participants during the study.

Carer impact will be measured using the ASCOT-carer[37], a validated measure of social-care related quality of life for carers, and the WEMWBS wellbeing scale.

See Table 1 for full details of trial assessments and timepoints.

Participant timeline

After participants express their interest in the study via an online form, they will be contacted by the research team to conduct a preliminary assessment of their eligibility. If potentially eligible, they will be invited to a baseline assessment. If they meet eligibility criteria and

consent to participate, they will become a trial participant. Randomisation will then be conducted within 2 weeks of the next group start date and communicated to the participant by email. Those enrolled in the study will receive updates from the study team such as blog posts. Follow-up assessments will be completed 8-, 16-, and 24- weeks after randomisation. Participants will be paid with a £15 voucher for each baseline and follow-up assessment they complete.

Sample size

The recruitment target is 70 participants from across England and Wales. A sample size of 70, with 80% follow-up rates, is deemed sufficient to inform a sample size calculation for a full RCT and to evaluate the rates of recruitment and retention with sufficient precision[38].

Recruitment

ATAG recruitment materials (e.g., posters and leaflets) will be shared electronically via charities (e.g. Ambitious about Autism, National Autistic Society, Autistica). Recruitment materials will also be shared via social media and traditional media resources (e.g., twitter, websites, press-releases), as well as various community organisations including charities and third sector providers of support services.

Potential participants will be identified within the NHS Participant Identification Centres (PICs) by clinical services staff. This can be done within the context of a clinical appointment or by clinician review of patient lists. Service staff may discuss the study with individuals during a consultation and provide them with an ATAG Study advert.

All potential participants will be referred to complete an ATAG Expression of Interest Form. The form can be completed by potential participants or those who support them via a secure online weblink, or via paper equivalent or on the telephone with a researcher if required. The aim of the expression of interest form is to ask individuals a short series of questions based on the inclusion/exclusion criteria. Those who do not meet the eligibility criteria will be informed of the outcome and no further data about the individual will be collected.

If the individual is potentially eligible to take part, additional information will be requested to allow for further eligibility assessment and participation. Those who are still potentially eligible will be invited to an online baseline assessment, and any accessibility needs or reasonable adjustments will be taken into consideration to allow access to this appointment.

The individual will be sent the baseline questionnaires via an online survey link in advance of this appointment. These appointments will be offered via video call as default, but can also be offered via voice only or phone call. The Baseline appointment is structured with an accessible PowerPoint presentation which potential participants receive in advance so that

they have time to prepare for the appointment. The eligibility criteria will be reviewed, the trial will be explained and the participant can ask any questions. At this point, there will be an assessment of risk to self and others following a risk standardised operating procedure. This involves completing the Q9 from the PHQ-9 (“Over the last two weeks, how often have you been bothered by thoughts that you would be better off dead, or of hurting yourself in some way?”). To assess risk to others, participants will be asked a series of questions developed for this study about risk to others. This will include asking about any incidents of school exclusion or contact with the police due to posing a risk to others. The participant will also be told about the proposed randomisation date and group start dates.

During this baseline discussion, the researcher will check that individuals are able to provide fully informed consent. This will be through asking a series of questions which check understanding of trial procedures; the right to withdrawal and that their involvement is completely voluntary. For example, to assess their ability to voluntarily consent, they will be asked “do you think you have to take part in this research?”.

For individuals who do not meet the eligibility criteria, their baseline questionnaire will be destroyed, and they will be offered a £15 gift voucher to recompense their time. If the researcher believes that the potential participant is fully eligible for the study, they will invite the participant to complete the consent form. Informed consent for both the trial (essential) and nested qualitative study (optional) will be captured via an eConsent (online) form.

If the baseline appointment is more than two weeks before randomisation will occur, the WEMWBS (primary outcome) will be sent to participants to complete in the two weeks before randomisation.

Allocation

The randomisation sequence will be generated by Sealed Envelope™. Randomisation will be stratified by age (16-17 or 18-25). Participants will be randomised to one of two treatment groups on a 1:1 ratio, that is either UYDY (intervention arm) or CAU (control arm).

The CI (or authorised delegate) will sign into the secure online randomisation system, enter the individual’s unique study I.D number and age; they will then receive the code that allocates the participant to the study treatment. The research team will inform the individual of their allocation, and securely share the contact details of those allocated to UYDY to the team facilitating the groups. The unblinded randomisation code will be held by selected members of the Trial Management Group (TMG).

Blinding

The Trial Management Group will be blinded to the allocation of treatment group, except for members of staff involved in data management, trial facilitators and supervisors of trial facilitators.

Data collection methods and data management

Source data for this trial consists of electronic versions of preliminary screening and expression of interest forms, consent form(s), participant and carer completed questionnaires and other records specific to the study. Data from all participants will be collected and retained in accordance with the UK Data Protection Act 2018 and UK General Data Protection Regulation 2018 (GDPR). Participants will be asked to consent to their personal information and research data being stored by the research team.

Standardised outcome instruments will be used throughout the trial; the components and timing of follow-up measures are shown in Table 1. All identifiable participant data will be entered into and stored on encrypted databases. All administrative and clinical study data will be stored in separate Questionpro forms. Questionpro is a secure, web-based electronic data capture (EDC) system designed for the collection of research data. The clinical data will be stored separately to the administrative data. Anonymised clinical data is linked by a study participant I.D. Data will be retained for at least 5-years after the end of the trial, and at the end of the archiving period, will be destroyed by confidential means with the exception of a final dataset which will be made available for data-sharing purposes.

Anonymous research data, including anonymised transcripts, will be stored securely and kept for future analysis with participant consent.

Participant retention will be targeted through sharing co-produced study blog posts to increase engagement.

Statistical methods

We will follow CONSORT guidelines throughout and present a CONSORT flowchart in the results. We will also report baseline characteristics of participants by arm.

To meet objective 1, assessing recruitment and retention, the percentage of those who are eligible and consent to be randomised will be calculated. Retention rates will be calculated as percentages of the number of participants in each arm who complete follow-up measures at each time point. All data will be used as randomised, and a percentage of missing data for each scale at each follow-up time point will be reported.

To meet objective 2, about the acceptability of randomisation and outcome measurement procedures. qualitative data is collected (see below).

For objective 3, CAU will be characterised by calculating the proportion of participants in CAU who receive each type of support.

For objective 4, the acceptability of the interventions will be assessed quantitatively by calculating the percentage of those who complete the intervention, the mean number of sessions completed (with three sessions considered the minimum acceptable number), and the standard deviation, as well as the number of adverse events in each arm.

For objective 5, outcome measure variances will be assessed with descriptive statistics, including means and standard deviations, calculated by intervention group and timepoint for each outcome measure. This will inform the power calculation for a fully-powered trial.

Carer impact sub-study

The carer data will also be analysed as described in objectives 1 and 5 above.

Qualitative study

We will conduct a qualitative study to meet aims 2 and 4, regarding the acceptability of randomisation and outcome measurement procedures and interventions (UYDY and CAU). Qualitative work with those who drop out of the study and those who continue attending will help us understand the factors contributing to any treatment drop-out.

All aspects of this part of the study (i.e., design, recruitment, interviewing, analysis, interpretation of findings, dissemination) will be conducted in partnership with autistic young people who are in the leadership team for the study.

All participants in the trial will be asked if they are willing to be contacted about taking part in an interview at the time of trial consent. Verbal consent will be taken at the point of interview for autistic young people and carers. Facilitators will complete written and audio consent at the point of interview.

Semi-structured one-to-one interviews will be conducted with 20 participants allocated to UYDY, 10 participants in CAU, 10 group facilitators, and 10 carers of participants. Purposive sampling will be undertaken, aiming for diversity of qualitative study participants with respect to ethnicity, gender, age, and geographical region, to ensure a range of viewpoints.

Interviews will be conducted online via video or audio-only calls. The topic guide will be developed with autistic young people and will cover areas including:

. Experiences of being recruited into the study

. Equipoise: whether participants had a preference for allocation to CAU or UYDY

. Experience of the interventions and follow-up assessments

. Suggestions for improvements to UYDY

. Suggestions on how to increase access and inclusion for minoritised participants (where applicable)

With informed consent, interviews will be recorded using an encrypted audio recorder for telephone interviews or the secure university approved online platform MS Teams or Zoom. Interviews with autistic participants will be conducted by an autistic young person wherever possible, and autism adaptations will be made depending on individual participant need. Adaptations will include to communication method (e.g. using the chat function where the individual is struggling to verbally communicate), environment (e.g. ensuring the individual has access to fidget toys where needed), and structure (e.g., having regular breaks where needed). Qualitative data collection and analysis will be conducted concurrently, with the topic guide being updated based on early interviews.

Qualitative Analysis

Interview recordings will be fully transcribed, anonymised, checked for accuracy and imported into NVivo qualitative data analysis software to aid data management.

Qualitative data will be analysed using Reflexive Thematic Analysis from a critical realist standpoint, as defined by Braun and Clarke[39]. Analysis will begin shortly after data collection starts, will be ongoing and iterative. Analysis will inform further data collection: for instance, analytic insights from data gathered in earlier interviews will help identify any changes that need to be made to the topic guide during later interviews.

The neurodiverse qualitative team will work together to conduct line-by-line coding of the transcripts and theme development to generate a robust analysis. A subset of transcripts will be analysed collaboratively in group meetings to ensure that a consistent approach to data analysis is undertaken. The team analysis will then be presented to the other co-applicants and stakeholder groups to ensure that the findings are understood as plausible, rigorous, and transferable.

Patient and Public Involvement

There has been significant patient and public involvement at all stages of this project. We are a neurodiverse team including autistic young people, researchers, and professionals. We have consulted various stakeholders in the design of the research and dissemination plans including autistic young people, a stakeholder group of professionals and parents, and a network of social care professionals. The intervention itself was also co-designed in a partnership between Ambitious about Autism and academic partners[27].

Risk and Adverse Events

A risk management standardised operating procedure has been developed for use by both research and social care staff throughout the trial. This will be implemented to ensure the safety of all participants if they express significant distress during the research process or a desire or intention to harm themselves or others.

Adverse Events (AEs) are expected throughout the course of this trial and will be recorded by the research team. These will be detected via study questionnaires and follow-up by the research team, as well as contact with the group facilitators for those allocated to UYDY. The research team will categorise whether adverse events are serious, expected, and related to trial participation. Serious adverse events are those that are life-threatening or result in death, require inpatient hospitalisation or prolonging existing hospitalisation, or result in persistent or significant disability or incapacity. Only non-serious AEs deemed to be linked to trial participation will be recorded. All serious AEs which are related to trial participation will be recorded and reported to the sponsor and Trial Steering Committee chair within 24 hours of the study team becoming aware of them.

Ethics and dissemination

Ethical approval for the study granted by the Health Research Authority and Wales REC 5 (Reference: 23/WA/0113). The trial was designed independently of the trial sponsor and funder. The trial will be monitored and audited in accordance with the Sponsor's policy, which is consistent with the UK Policy Framework for Health and Social Care Research. Modifications to the trial procedures will be reported to all relevant parties and appropriate ethical approval will be sought for such changes. Fully informed consent will be gained from all participants; to ensure that individuals have read and understood the information sheet, researchers will ask them to summarise their understanding of trial procedures, the voluntary nature of participation, and right to withdraw, before the consent form is presented (see Supplemental Material).

The combined Data Monitoring and Trial Steering Committee is independent of the sponsor and no competing interests have been reported. It includes experts in child mental health trials, autism intervention and research, and an independent statistician.

Dissemination

An engagement plan will be produced with the TMG, collaborators and members of the Patient Advisory Group. We will seek input from the Trial Steering Committee about the suitability of engagement plans and the dissemination policy.

In terms of the overall study findings, we will produce papers in peer-reviewed journals, presentations and blogs, sharing findings both in academic settings (conferences) and with the autism community (stakeholder events), as well as policy makers and professionals. Our

results will inform the design of a full RCT. Authors of all publications will meet the ICMJE criteria for authorship.

Author contributions: All authors contributed to the conceptualisation, methodology and writing – review and editing. The first author is the guarantor.

Funding statement: This work was supported by the National Institute for Health Research and Autistica grant number NIHR204276.

Competing interests: None to declare

Table 2.

**Primary registry ID number**	ISRCTN10513626	
**Date of registration**	5th October 2023	
**Funder**	National Institute for Health Research – Research for Social Care and Autistica (NIHR204276)	
**Sponsor**	University of Bath (UniBath)	
**Contact for all enquiries**	atag@bath.ac.uk	
**Protocol version number**	V1.4	
**Public title**	Autism Transition to Adulthood Group	
**Scientific title**	Peer-group intervention to promote successful transition to adulthood for autistic people: A feasibility RCT of the Autism Transition to Adulthood Group (ATAG)	
**Countries of Recruitment**	England and Wales	
**Interventions**	1. Understanding You, Discovering You (UYDY) groups<br>2. Care as Usual (CAU)	
**Inclusion criteria**	• Age between 16-25 years.<br>• A clinical diagnosis of Autism Spectrum Disorder (ASD)	
**Exclusion criteria**	• Risk of suicide<br>• Risk of harm to others<br>• Have attended autism psychoeducation or post-diagnostic support with a professional over the past 12 months<br>• English, non-English & Welsh literacy levels such that the intervention materials are inaccessible without reasonable adjustments and a supporting person is not available	
**Trial design**	Feasibility randomised controlled trial with two arms, UYDY and CAU	
**Date of first enrolment**	December 2023	
**Sample size**	70	
**Recruitment status**	Recruiting	
	**Objectives**	**Outcome Measures**
	1. Assess the rates of recruitment and retention to inform the design of a full-scale RCT	1. Warwick-Edinburgh Mental Wellbeing Scale (WEMWBS)

	2. Assess the acceptability of randomisation and outcome measurement procedures to participants	2. Autism social identification measure<br>3. EQ-5D-5L<br>4. Interpersonal Support Evaluation List – short<br>5. UCLA loneliness scale<br>6. Use of services questionnaire based on the Client Service Receipt Inventory<br>7. Carer measures: WEMWBS & ASCOT-carer
	3. Characterise Care As Usual (CAU)	
	4. Assess the acceptability of the A-TAG intervention and CAU	
	5. Calculate outcome measure variances for use in a power calculation for a full trial	
**Ethics Review**	Ethical approval for the study granted by the Health Research Authority and Wales REC 5 (Reference: 23/WA/0113)	
**Study duration**	• Funding start date: 01 September 2023<br>• Anticipated duration: 22 months *(total; subject to change)*<br>• Anticipated end date: 30 June 2025 *(subject to change)*	
**IDP sharing statement**	Participants are asked whether they consent to their anonymised data being shared with other researchers who have received appropriate ethical approval for their research. This will be made available to other eligible researchers on request.	

References

[1]American Psychiatric Association. Diagnostic and Statistical Manual of Mental Disorders. 5th ed. 2013.

[2] Hirvikoski, T., Mittendorfer-Rutz, E., Boman, M., Larsson, H., Lichtenstein, P., & Bölte, S. (2016). Premature mortality in autism spectrum disorder. *The British Journal of Psychiatry*, *208*(3), 232-238.

[3] Howlin, P., Moss, P., Savage, S., & Rutter, M. (2013). Social outcomes in mid-to later adulthood among individuals diagnosed with autism and average nonverbal IQ as children. *Journal of the American Academy of Child & Adolescent Psychiatry*, *52*(6), 572-581.

[4] Lai, M. C., Kassee, C., Besney, R., Bonato, S., Hull, L., Mandy, W., ... & Ameis, S. H. (2019). Prevalence of co-occurring mental health diagnoses in the autism population: a systematic review and meta-analysis. *The Lancet Psychiatry*, *6*(10), 819-829.

[5] Rydzewska, E., Hughes-McCormack, L. A., Gillberg, C., Henderson, A., MacIntyre, C., Rintoul, J., & Cooper, S. A. (2019). Prevalence of sensory impairments, physical and intellectual disabilities, and mental health in children and young people with self/proxy-reported autism: Observational study of a whole country population. *Autism, 23*(5), 1201-1209.

[6] van Agteren, J., Iasiello, M., Lo, L., Bartholomaeus, J., Kopsaftis, Z., Carey, M., & Kyrios, M. (2021). A systematic review and meta-analysis of psychological interventions to improve mental wellbeing. *Nature human behaviour*, *5*(5), 631-652.

[7] Iasiello, M., van Agteren, J., Keyes, C. L., & Cochrane, E. M. (2019). Positive mental health as a predictor of recovery from mental illness. *Journal of Affective Disorders*, *251*, 227-230.

[8] Sawyer, S. M., Azzopardi, P. S., Wickremarathne, D., & Patton, G. C. (2018). The age of adolescence. *The lancet child & adolescent health*, *2*(3), 223-228.

[9] Moser, C., Smith DaWalt, L., Burke, M. M., & Taylor, J. L. (2024). Emerging adulthood in autism: Striving for independence or interdependence?. *Autism*, 13623613241245647.

[10] Levy, A., & Perry, A. (2011). Outcomes in adolescents and adults with autism: A review of the literature. *Research in Autism Spectrum Disorders*, *5*(4), 1271-1282.6.

[11] Crane, L., Batty, R., Adeyinka, H., Goddard, L., Henry, L. A., & Hill, E. L. (2018). Autism diagnosis in the United Kingdom: Perspectives of autistic adults, parents and professionals. *Journal of autism and developmental disorders*, *48*(11), 3761-3772.

[12] Scatting, M. L., Micai, M., Ciaramella, A., Salvitti, T., Fulceri, F., Fatta, L. M., ... & Schendel, D. (2021). Real-world experiences in autistic adult diagnostic services and post-diagnostic support and alignment with services guidelines: Results from the ASDEU study. *Journal of Autism and Developmental Disorders*, 1-18.

[13] *SUPPORT SURROUNDING DIAGNOSIS An Inquiry into Pre-and Post- Support for the Autism Diagnosis Pathway*. (n.d.). Retrieved June 7, 2024, from https://irp.cdn-website.com/79f83829/files/uploaded/westminster-commission-on-autism-report-support-surrounding-diagnosis.pdf

[14] Beresford, B. A., Mukherjee, S. K. M., Mayhew, E. T., Park, A. L., Stuttard, L., Allgar, V. L., & Knapp, M. (2020). Evaluating specialist autism teams' provision of care and support for autistic adults without learning disabilities: the SHAPE mixed-methods study. *Health Services and Delivery Research*.

[15] Backman, A., Mellblom, A., Norman-Claesson, E., Keith-Bodros, G., Frostvittra, M., Bölte, S., & Hirvikoski, T. (2018). Internet-delivered psychoeducation for older adolescents and young adults with autism spectrum disorder (SCOPE): an open feasibility study. *Research in Autism Spectrum Disorders*, *54*, 51-64.

[16] Nadig, A., Flanagan, T., White, K., & Bhatnagar, S. (2018). Results of a RCT on a Transition Support Program for Adults with ASD: Effects on Self-Determination and Quality of Life. *Autism research*, *11*(12), 1712-1728.

[17] Crane, L., Hearst, C., Ashworth, M., Davies, J., & Hill, E. L. (2021). Supporting newly identified or diagnosed autistic adults: An initial evaluation of an autistic-led programme. *Journal of autism and developmental disorders*, *51*(3), 892-905.

[18] Crane, L., Hearst, C., Ashworth, M., & Davies, J. (2023). Evaluating the online delivery of an autistic-led programme to support newly diagnosed or identified autistic adults. *Autism & Developmental Language Impairments*, *8*, 23969415231189608.

[19] Shea, L. L., Wong, M. Y., Song, W., Kaplan, K., Uppal, D., & Salzer, M. S. (2024). Autistic-delivered peer support: A feasibility study. *Journal of Autism and Developmental Disorders*, *54*(2), 409-422.

[20] Valentine, A. Z., Hall, S. S., Young, E., Brown, B. J., Groom, M. J., Hollis, C., & Hall, C. L. (2021). Implementation of telehealth services to assess, monitor, and treat neurodevelopmental disorders: systematic review. *Journal of Medical Internet Research*, *23*(1), e22619.

[21] Erikson, E. H. (1950). *Childhood and society* (1st ed.). Norton & Company.

[22] Marcia, J. E. (1966). Development and validation of ego-identity status. *Journal of Personality and Social Psychology*, **3**(5), 551–558. https://doi.org/10.1037/h0023281

[23] Gray, S. M., McMorris, C. A., Mudry, T. E., & McCrimmon, A. W. (2024). An exploration of diagnostic identity for autistic individuals: A systematic review of existing literature. *Research in Autism Spectrum Disorders*, *114*, 102394.

[24] Davies, J., Cooper, K., Killick, E., Sam, E., Healy, M., Thompson, G., ... & Crane, L. (2024). Autistic identity: A systematic review of quantitative research. *Autism Research*.

[25] Haar, T., Brownlow, C., Hall, G., Heyworth, M., Lawson, W., Poulsen, R., ... & Pellicano, E. (2024). 'We have so much to offer': Community members' perspectives on autism research. *Autism*, 13623613241248713.

[26] Chan A-W, Tetzlaff JM, Gøtzsche PC, Altman DG, Mann H, Berlin J, Dickersin K, Hróbjartsson A, Schulz KF, Parulekar WR, Krleža-Jerić K, Laupacis A, Moher D. SPIRIT 2013 Explanation and Elaboration: Guidance for protocols of clinical trials. BMJ. 2013;346:e7586

[27] Davies, J., Redmayne, B., Allain, L., Portway, L., Mandy, W., Cooper, K., & Crane, L. (In Press). The co-design and initial evaluation of a peer support program for autistic young adults. *Autism in Adulthood.*

[28] Trousselard, M., Steiler, D., Dutheil, F., Claverie, D., Canini, F., Fenouillet, F., … Franck, N. (2016). Validation of the Warwick–Edinburgh Mental Well-Being Scale (WEMWBS) in French psychiatric and general populations. *Psychiatry Research, 245*, 282–290.

[29] Hull, L., Mandy, W., Lai, M. C., Baron-Cohen, S., Allison, C., Smith, P., & Petrides, K. V. (2019). Development and validation of the camouflaging autistic traits questionnaire (CAT-Q). *Journal of Autism and Developmental Disorders*, *49*(3), 819-833.

[30] Leach CW, Van Zomeren M, Zebel S, Vliek ML, Pennekamp SF, Doosje B, Ouwerkerk JW, Spears R. Group-level self-definition and self-investment: a hierarchical (multicomponent) model of in-group identification. Journal of personality and social psychology. 2008 Jul;95(1):144.

[31] Cooper, K., Smith, L. G., & Russell, A. (2017). Social identity, self-esteem, and mental health in autism. *European Journal of Social Psychology*, *47*(7), 844-854.

[32] Maitland CA, Rhodes S, O'Hare A, Stewart ME. Social identities and mental well-being in autistic adults. Autism. 2021 Aug;25(6):1771-83.

[33] Russell, A., Gaunt, D. M., Cooper, K., Barton, S., Horwood, J., Kessler, D., ... & Wiles, N. (2020). The feasibility of low-intensity psychological therapy for depression co-occurring with autism in adults: The Autism Depression Trial (ADEPT)–a pilot randomised controlled trial. *Autism*, *24*(6), 1360-1372.

[34] Cohen S., Mermelstein R., Kamarck T., & Hoberman, H.M. (1985). Measuring the functional components of social support. In Sarason, I.G. & Sarason, B.R. (Eds), Social support: theory, research, and applications. The Hague, Netherlands: Martinus Niijhoff.

[35] Russell, D., Peplau, L. A., & Cutrona, C. E. (1980). The revised UCLA Loneliness Scale: concurrent and discriminant validity evidence. Journal of personality and social psychology, 39(3), 472.

[36] Beecham, J., & Knapp, M. (1995). The client service receipt inventory. Discussion Paper 1492. Personal Social Services Research Unit, University of Kent at Canterbury.

[37] Rand, S. E., Malley, J. N., Netten, A. P., & Forder, J. E. (2015). Factor structure and construct validity of the adult social care outcomes toolkit for carers (ASCOT-carer). *Quality of Life Research*, *24*(11), 2601-2614.

[38] Teare, M. D., Dimairo, M., Shephard, N., Hayman, A., Whitehead, A., & Walters, S. J. (2014). Sample size requirements to estimate key design parameters from external pilot randomised controlled trials: a simulation study. Trials, 15(1), 1-13.

[39] Braun, V., & Clarke, V. (2006). Using thematic analysis in psychology. *Qualitative research in psychology*, *3*(2), 77-101.

SPIRIT checklist: ATAG study

		Reporting Item	Page Number
**Administrative information**			
Title	#1	Descriptive title identifying the study design, population, interventions, and, if applicable, trial acronym	1
Trial registration	#2a	Trial identifier and registry name. If not yet registered, name of intended registry	16
Trial registration: data set	#2b	All items from the World Health Organization Trial Registration Data Set	16
Protocol version	#3	Date and version identifier	16
Funding	#4	Sources and types of financial, material, and other support	16
Roles and responsibilities: contributorship	#5a	Names, affiliations, and roles of protocol contributors	1
Roles and responsibilities: sponsor contact information	#5b	Name and contact information for the trial sponsor	16
Roles and responsibilities: sponsor and funder	#5c	Role of study sponsor and funders, if any, in study design; collection, management, analysis, and interpretation of data; writing of the report; and the decision to submit the report for publication, including whether they will have ultimate authority over any of these activities	14
Roles and responsibilities: committees	#5d	Composition, roles, and responsibilities of the coordinating centre, steering committee, endpoint adjudication committee, data management team, and other individuals or groups overseeing the trial, if applicable (see Item 21a for data monitoring committee)	14
**Introduction**			
Background and rationale	#6a	Description of research question and justification for undertaking the trial, including summary of relevant studies (published and unpublished)	3-5

		examining benefits and harms for each intervention	
Background and rationale: choice of comparators	#6b	Explanation for choice of comparators	6-7
Objectives	#7	Specific objectives or hypotheses	5
Trial design	#8	Description of trial design including type of trial (eg, parallel group, crossover, factorial, single group), allocation ratio, and framework (eg, superiority, equivalence, non-inferiority, exploratory)	5
**Methods: Participants, interventions, and outcomes**			
Study setting	#9	Description of study settings (eg, community clinic, academic hospital) and list of countries where data will be collected. Reference to where list of study sites can be obtained	5
Eligibility criteria	#10	Inclusion and exclusion criteria for participants. If applicable, eligibility criteria for study centres and individuals who will perform the interventions (eg, surgeons, psychotherapists)	5-6
Interventions: description	#11a	Interventions for each group with sufficient detail to allow replication, including how and when they will be administered	6-7
Interventions: modifications	#11b	Criteria for discontinuing or modifying allocated interventions for a given trial participant (eg, drug dose change in response to harms, participant request, or improving / worsening disease)	n/a
Interventions: adherance	#11c	Strategies to improve adherence to intervention protocols, and any procedures for monitoring adherence (eg, drug tablet return; laboratory tests)	7
Interventions: concomitant care	#11d	Relevant concomitant care and interventions that are permitted or prohibited during the trial	7
Outcomes	#12	Primary, secondary, and other outcomes, including the specific measurement variable (eg, systolic blood pressure), analysis metric (eg,	9

		change from baseline, final value, time to event), method of aggregation (eg, median, proportion), and time point for each outcome. Explanation of the clinical relevance of chosen efficacy and harm outcomes is strongly recommended	
Participant timeline	#13	Time schedule of enrolment, interventions (including any run-ins and washouts), assessments, and visits for participants. A schematic diagram is highly recommended (see Figure)	9
Sample size	#14	Estimated number of participants needed to achieve study objectives and how it was determined, including clinical and statistical assumptions supporting any sample size calculations	10
Recruitment	#15	Strategies for achieving adequate participant enrolment to reach target sample size	10-11
**Methods: Assignment of interventions (for controlled trials)**			
Allocation: sequence generation	#16a	Method of generating the allocation sequence (eg, computer-generated random numbers), and list of any factors for stratification. To reduce predictability of a random sequence, details of any planned restriction (eg, blocking) should be provided in a separate document that is unavailable to those who enrol participants or assign interventions	11
Allocation concealment mechanism	#16b	Mechanism of implementing the allocation sequence (eg, central telephone; sequentially numbered, opaque, sealed envelopes), describing any steps to conceal the sequence until interventions are assigned	11
Allocation: implementation	#16c	Who will generate the allocation sequence, who will enrol participants, and who will assign participants to interventions	11
Blinding (masking)	#17a	Who will be blinded after assignment to interventions (eg, trial participants, care	11

		providers, outcome assessors, data analysts), and how	
Blinding (masking): emergency unblinding	#17b	If blinded, circumstances under which unblinding is permissible, and procedure for revealing a participant’s allocated intervention during the trial	n/a
**Methods: Data collection, management, and analysis**			
Data collection plan	#18a	Plans for assessment and collection of outcome, baseline, and other trial data, including any related processes to promote data quality (eg, duplicate measurements, training of assessors) and a description of study instruments (eg, questionnaires, laboratory tests) along with their reliability and validity, if known. Reference to where data collection forms can be found, if not in the protocol	11-12
Data collection plan: retention	#18b	Plans to promote participant retention and complete follow-up, including list of any outcome data to be collected for participants who discontinue or deviate from intervention protocols	12
Data management	#19	Plans for data entry, coding, security, and storage, including any related processes to promote data quality (eg, double data entry; range checks for data values). Reference to where details of data management procedures can be found, if not in the protocol	11-12
Statistics: outcomes	#20a	Statistical methods for analysing primary and secondary outcomes. Reference to where other details of the statistical analysis plan can be found, if not in the protocol	12-13
Statistics: additional analyses	#20b	Methods for any additional analyses (eg, subgroup and adjusted analyses)	13
Statistics: analysis population and missing data	#20c	Definition of analysis population relating to protocol non-adherence (eg, as randomised analysis), and any statistical methods to handle missing data (eg, multiple imputation)	12
**Methods: Monitoring**			

Data monitoring: formal committee	#21a	Composition of data monitoring committee (DMC); summary of its role and reporting structure; statement of whether it is independent from the sponsor and competing interests; and reference to where further details about its charter can be found, if not in the protocol. Alternatively, an explanation of why a DMC is not needed	14
Data monitoring: interim analysis	#21b	Description of any interim analyses and stopping guidelines, including who will have access to these interim results and make the final decision to terminate the trial	n/a
Harms	#22	Plans for collecting, assessing, reporting, and managing solicited and spontaneously reported adverse events and other unintended effects of trial interventions or trial conduct	14-15
Auditing	#23	Frequency and procedures for auditing trial conduct, if any, and whether the process will be independent from investigators and the sponsor	14
**Ethics and dissemination**			
Research ethics approval	#24	Plans for seeking research ethics committee / institutional review board (REC / IRB) approval	14
Protocol amendments	#25	Plans for communicating important protocol modifications (eg, changes to eligibility criteria, outcomes, analyses) to relevant parties (eg, investigators, REC / IRBs, trial participants, trial registries, journals, regulators)	14
Consent or assent	#26a	Who will obtain informed consent or assent from potential trial participants or authorised surrogates, and how (see Item 32)	10
Consent or assent: ancillary studies	#26b	Additional consent provisions for collection and use of participant data and biological specimens in ancillary studies, if applicable	13
Confidentiality	#27	How personal information about potential and enrolled participants will be collected, shared, and maintained in order to protect confidentiality before, during, and after the trial	11-12

Declaration of interests	#28	Financial and other competing interests for principal investigators for the overall trial and each study site	15
Data access	#29	Statement of who will have access to the final trial dataset, and disclosure of contractual agreements that limit such access for investigators	17
Ancillary and post trial care	#30	Provisions, if any, for ancillary and post-trial care, and for compensation to those who suffer harm from trial participation	n/a
Dissemination policy: trial results	#31a	Plans for investigators and sponsor to communicate trial results to participants, healthcare professionals, the public, and other relevant groups (eg, via publication, reporting in results databases, or other data sharing arrangements), including any publication restrictions	15
Dissemination policy: authorship	#31b	Authorship eligibility guidelines and any intended use of professional writers	15
Dissemination policy: reproducible research	#31c	Plans, if any, for granting public access to the full protocol, participant-level dataset, and statistical code	17
**Appendices**			
Informed consent materials	#32	Model consent form and other related documentation given to participants and authorised surrogates	n/a
Biological specimens	#33	Plans for collection, laboratory evaluation, and storage of biological specimens for genetic or molecular analysis in the current trial and for future use in ancillary studies, if applicable	n/a

The SPIRIT Explanation and Elaboration paper is distributed under the terms of the Creative Commons Attribution License CC-BY-NC. This checklist was completed on 09. June 2024 using https://www.goodreports.org/, a tool made by the EQUATOR Network in collaboration with Penelope.ai
